# Chronic exposure to emissions from photocopiers in copy shops causes oxidative stress and systematic inflammation among photocopier operators in India

**DOI:** 10.1186/1476-069X-12-78

**Published:** 2013-09-11

**Authors:** Nithya Elango, Vallikkannu Kasi, Bhuvaneswari Vembhu, Jeyanthi Govindasamy Poornima

**Affiliations:** 1 Department of Biochemistry, Biotechnology and Bioinformatics Avinashilingam Institute for Home Science and Higher Education for Women Coimbatore, Tamilnadu, India

**Keywords:** Photocopiers, Particulate matter, Biomarkers, Lung function, Occupational exposure, Toner

## Abstract

**Background:**

We assessed indoor air quality in photocopier centers and investigated whether occupational exposure to emissions from photocopiers is associated with decline in lung function or changes in haematological parameters, oxidative stress and inflammatory status.

**Methods:**

Indoor air quality was monitored in five photocopier centers. Pulmonary function was assessed by spirometry in 81 photocopier operators (64 male and 17 female) and 43 healthy control (31 male and 12 female) subjects. Hematological status, serum thio-barbituric acid reactive substances (TBARS), total ferric reducing antioxidant capacity (FRAC), leukotriene B_4_ (LTB_4_), 8-isoprostane, C reactive protein (CRP), interleukin 8 (IL-8), clara cell protein (CC-16), intercellular adhesion molecule 1 (ICAM-1) and eosinophilic cationic protein (ECP) were analyzed. Relationships between cumulative exposure, lung function and inflammatory markers were assessed.

**Results:**

PM_10_ and PM_2.5_ were above the permissible levels in all the photocopier centers, whereas the levels of carbon monoxide, nitrogen oxides, ozone, sulphur dioxide, lead, arsenic, nickel, ammonia, benzene and benzo(a)pyrene were within Indian ambient air quality standards. Lung function was similar in the photocopier operators and control subjects. Serum TBARS was significantly higher and FRAC was lower among photocopier operators when compared to healthy controls. Plasma IL-8, LTB_4_, ICAM-1 and ECP were significantly higher in the photocopier exposed group.

**Conclusions:**

Photocopiers emit high levels of particulate matter. Long term exposure to emissions from photocopiers was not associated with decreased lung function, but resulted in high oxidative stress and systemic inflammation leading to high risk of cardiovascular diseases.

## Background

Photocopiers are considered as essential amenities at offices and business establishments. The world printing machinery and supplies industry is expected to exceed $21 billion by 2015. In the last two years alone, more than 250 million photocopiers have been sold all over the world. The market is driven by demand for digital colour presses, specialty printers and inkjet printers. The changing landscape of technology also fuels the printing machinery and supplies industry with new products, innovation and dynamic media giving the market a significant boost [1]. Consequently, the number of people operating photocopiers around the world may run into millions.

Despite the advantages and commercial benefits of photocopiers, they are also sources of air pollution. During operation, photocopiers emit toner particles, toxic gases namely ozone, nitrogen dioxide, volatile organic compounds, semi-volatile organic compounds, radiation, particulate matter, paper particles, nano particles and extremely low-frequency electromagnetic fields [2-4].

Till this date, health studies on occupational exposure to emissions from photocopiers are inconclusive and not comprehensive. Several studies have associated chronic exposure to emissions from photocopiers in occupational settings with symptoms like breathlessness, non-allergic rhinitis, sore throat, cough, asthma, pseudo allergic inflammation of the respiratory tract, upper respiratory tract infections, skin and eye irritations, headache, sick building syndrome, siderosilicosis, granulomotous pneumonitis and sarcoidosis [5-9]. A number of authors have reported elevated DNA damage among operators in photocopier centers [10-13]. However, others [2] suggested current exposure levels in photocopier centres may be sufficiently safe in well controlled work environments.

Photocopier toners are chief components of photocopier emissions. The effects of toner exposure have been extensively studied in cell culture, animal models and operators in toner manufacturing units and delineate contradictory results. Studies [14] in cell lines reported that individual toners are genotoxic. Animal studies found toners to be toxicologically inert and non carcinogenic [15,16]. However, intra-tracheal instillation of very high doses of toner powder produced significantly increased lung tumor rates in rats [17-19].

Sub-mesothelial deposition of carbon nanoparticles in the peritoneum of toner dust exposed worker was reported [20]. When respiratory health of workers in toner manufacturing units were assessed, studies suggested deterioration of respiratory health related to toner dust exposure was less likely to occur in current well controlled work environments [21-23].

In this study we assessed indoor air quality in photocopier centers. We investigated, in a cross sectional design, whether occupational exposure to emissions from photocopiers is associated with impaired lung function and/or induce changes in haematological, oxidative and inflammatory status. This study was carried out in Coimbatore, a metropolitan city in south India between January 2011 and February 2012.

## Materials and methods

### Air quality monitoring

Indoor air quality was monitored in selected photocopier centers as per Indian Standard guidelines (IS 5182) [24]. Air sampling was conducted at 5 photocopy centers in Coimbatore, during September 2012. Table 1 lists the methods used to assess the different various air quality parameters.

**Table 1:** Methods followed to assess indoor air quality in photocopier centers

**Air quality parameters**	**Air flow rate**	**Method**
Carbon monoxide	40 mL/minute for 1 hour	Indicator tube using silicomolybdate [25]
Nitrogen dioxide	200 mL/minute for 4 hours	Spectrophotometric method [26,27]
Ozone	1 L/minute for 1 hour	Chemical method [28]
Fine particulate matter (PM_2.5_)	1.5 L/minute for 8 hours	Gravimetric method [29]
Particulate matter (PM_10_)	1000 L/minute for 8 hours	Gravimetric method [27,30]
Sulphur dioxide	1 litre/minute for 30 minutes	West and Gaeke method [27,31]
Lead, Arsenic, Nickel	1 litre/minute for 8 hours	Atomic Atomic Spectroscopy after absorption on filter paper [30,32]
Ammonia	1 litre/minute for 1 hour	Indophenol method [27]
Benzene, Benzo(a)pyrene	1.2 ml/minute	Active sampling by activated charcoal tube, desorbed by Carbondisulphide followed by Gas chromatography with phenyl dimethyl polysiloxane column and flame ionisation detector [33,34]

Measurements were made on working days for a period of 8 hours by an ISO 2001 certified commercial laboratory. Indoor air quality assessment was carried out by positioning the sampling equipment directly opposite to the photocopier(s) in all the centers. In Coimbatore, most photocopy centers are located at ground level. The width of each photocopy center is approximately 10 meters. A typical photocopier center consists of a concrete building with walls on three sides. The fourth side consists of a retractable shutter which is closed only during out of business hours. Typical interior materials used in photocopy centers include cement floor, painted concrete ceiling, painted concrete walls and metal shutters. Usually some metal desks, chairs, one to two computers, laminating and binding machines, are present in the confined space of a photocopy center. Basic information of the photocopy centers, including room dimensions, environmental conditions, business hours, types of ventilation, number of photocopiers, number of copies made on an average work day and the presence of other VOC-emitting sources were collected. Table 2 lists the physical characteristics of the photocopy centers. Table 3 lists the number of photocopiers present in each copy shop and the details about the toners used. Facsimile machines, color photocopiers and laser printers were also seen in some centers, but their use was very rare. Therefore, the current study focused on the emission from black-and-white photocopiers.

**Table 2:** Physical characteristics of photocopy centers where indoor air quality was measured

**Copy center**	**Copy center volume (m**^**3**^**)**	**Ventilation**	**Other VOC sources**	**Flooring**	**Roof**	**Walls**	**Doors**	**Other furniture**	**No of entrances**
1	1200	One way opening	Computer (1), Ink jet printer (1)	Cement	Cement	Cement	Front Metal Shutter	Table-1, Chair-1	1
2	1600	One way opening	Printer (1), Carpet (1), Fax Machine (1), Incense (1), Scanner (1), Computer (3) Carpet (1)	Cement with Vinyl sheet	Cement	Cement	Front Metal Shutter	Table-2, Chair - 2, Revolving Chair- 7	1
3	960	2-way opening	Computer (3), Paper cutting (1), Printer (2)	Marble	Cement, Varnish	Cement, Oil painted	Front/Back Metal Shutters	Table-1, Chair - 3	2
4	4800	3-way opening	Computer (1), Printer (1), Incense (1), Binding Machine (1), Photocopiers under repair (3)	Mosaic	Cement	Painted	Front Metal Shutter	Computer Table-1, Chair - 3, Table-1	2
5	4800	1-way opening	Fascimile machine (1), Binding Machine (1) Computer (1), Printer (1) Photocopiers under repair (2)	Cement	Cement	Painted	Front Metal Shutter	Computer Table-2, Chair - 2	1

**Table 3:** Characteristics of photocopiers and toners used in the photocopy centers

**Copy center**	**Business hours**	**Toner cake**	**Toner category**	**Average No of copies/day**	**Number of copy machines**	**Type of copy machines**	**Make of copy machines**	**Copy speed**
1	8 a.m.- 8 p.m.	ITDL	Dry, Wet	1500	2	Black (1)	Canon 3530	60/min
						Colour (1)	Canon 6000	42/min
2	8 a.m.- 9 p.m	CFI, ITDL	Dry, Wet	1500	2	Black (1)	Canon 3530	60/min
						Colour (1)	Canon 6000	42/min
3	8 a.m.- 10 p.m	PCS	Dry, Wet	7000	4	Black (3)	a. IR105 (2) b. Canon 6570	a.105/min b. 65/min
						Colour (1)	IR 3200	32/min
4	9 a.m.- 9 p.m.	ITDL	Dry	3000	2	Black	Canon 6000	60/min
5	8 a.m - 9 p.m.	Jet Black	Dry	5000	1	Black	IR 6060	60/min

### Study population

The eligible population in the selected region consisted of photocopier operators who had worked in photocopier centers for at least five years. People were invited to participate in the program by individual approach. Free blood tests and pulmonary function tests, were proposed to volunteer subjects with occupational exposure to emissions from photocopiers. All participants were actively working at the time of the study. All the subjects of the study were confined to the same geographical area to minimize interferences from ambient air pollution. All interviews, pulmonary function tests and blood collection were carried out at the photocopier centers where the subjects worked between 10.00 a.m. and 2.00 p.m. on a working day in a single visit. This study was approved by the Human Ethical Committee of Avinashilingam Institute for Home Science and Higher Education for Women, Coimbatore (HEC.2011.24). Subjects gave informed consent before data and sample collection.

### Selection of subjects

Using a standardized interview schedule, personal, socio economic, professional and general health details were collected during in person interviews. Exposure to photocopiers (in terms of years of occupation at photocopier centers, average working hours/day, average working days/week), smoking histories, exposure to second hand tobacco smoke and fuels used were recorded in the interview schedule. Based on St. George’s respiratory questionnaire [35], a respiratory symptoms questionnaire was also included in the interview schedule. Subjects with any known ailments like cardiovascular diseases, asthma, diabetes mellitus, hypertension, hyperthyroidism, epilepsy, etc., were excluded from the study. None of the selected subjects were on regular medications.

### Photocopier exposed group

Occupational exposure was defined as minimum five years employment in a xerographic unit. Male and female operators in the age group of 20 to 60 years with at least 5 years exposure to emissions from photocopiers were included in the exposed group. Cumulative exposure to photocopiers was calculated from the number of working hours/day and number of working days/week. Exposure was assessed by the amount of time spent in photocopier centers.

No. of hours exposed = No. of working hours/day × No. of working days/week × No. of years of exposure × 50 weeks/year.

### Control group

Healthy male and female subjects with no professional exposure to emissions from photocopiers in the age group of 20 – 60 years were categorized as control group. Subjects in this group consisted of clerks, cooks, drivers, housewives, shop keepers, etc.

### Pulmonary function test

Pulmonary function was assessed by using Vitalograph Alpha 6000, UK. Spirometry was performed according to the American Thoracic Society guidelines [36]. Parameters used for analysis of the flow–volume curve were forced vital capacity (FVC), forced expiratory volume in 1 second (FEV_1_), FEV_1_/FVC ratio, peak expiratory flow (PEF) and maximum mid expiratory flow (FEF_25-75_). Results were expressed as percentages of predicted values, using equations published in 1993 by the European Respiratory Society.

### Analysis of biomarkers

Peripheral venous blood samples were collected. Plasma/serum were separated, aliquoted into labeled cryo vials and stored at -80°C.

Lipid peroxidation and total antioxidant capacity were measured in serum [37,38]. The following markers were assessed in EDTA plasma by commercial ELISA kits mentioned: LTB_4_, 8-Isoprostane, CRP and glutathione peroxidase - Cayman Chemical, USA; IL-8 - Koma Biotech, Korea; ICAM-1, CC-16 and ECP - USCN Life Sciences, China. Myeloperoxidase was estimated in lithium heparin plasma by ELISA kit from Enzo Life Sciences, Switzerland. Intra-assay co-efficients of variations for the above ELISA kits varied from 1.4% to 4.0% and inter-assay co-efficients of variations were between 2.4% and 4.4%.

### Statistical analysis

Demographic variables were compared between the two study groups using analysis of variance (for continuous variables) and Chi-square test (for categorical variables). Odds ratio was used to assess the incidence of respiratory problems among the two groups. Differences between two groups were assessed using student’s *t* test (normal data) or Mann Whitney test (non-normal distribution). Correlations were assessed using the Spearman’s rank correlation. Statistical significance was reported at two tailed p < 0.05. SPSS version 16.0 was used for statistical analysis and drawing graphs.

## Results

### Indoor air quality in photocopier centers

Indoor air quality was assessed in a representative sample of five photocopier centers (Table 4).

**Table 4:** Indoor air quality in select photocopier centers in Coimbatore

**Air quality parameters**	**Background levels (n = 5)**	**Mean levels (n = 5)**	**Indoor air quality**	**Reference levels (NAAQS, 2009)**
Carbon monoxide (CO) (mg/m^3^)	<1.2	<1.2	<1.2	2.0
Nitrogen dioxide (NO_2_) (μg/m^3^)	8.0 ± 2.6	8.4 ± 3.8	9.1	80
Ozone (O_3_) (μg/m^3^)	<9.8	<9.8	<9.8	100
Fine particulate matter (PM_2.5_) (μg/m^3^)	128.1 ± 21.4	168.2 ± 34.6	78.5	60
Particulate matter (PM_10_) (μg/m^3^)	241.7 ± 44.2	376.4 ± 63.8	106.7	100
Sulphur dioxide (SO_2_) (μg/m^3^)	3.5 ± 1.5	4.7 ± 2.3	5.1	80
Lead (Pb) (μg/m^3^)	<0.1	<0.1	<0.1	1.0
Arsenic (As) (ng/m^3^)	<0.1	<0.1	<0.1	6.0
Nickel (Ni) (ng/m^3^)	<0.1	<0.1	<0.1	20
Ammonia (NH_3_) (μg/m^3^)	2.3	2.5	2.7	400
Benzene (C_6_H_6_) (μg/m^3^)	<1.0	<1.0	<1.0	5.0
Benzo(a)pyrene (BaP) (ng/m^3^)	<1.0	<1.0	<1.0	1.0

*NAAQS* National Ambient Air Quality Standards, 2009.

*Mg* Milligram, *μg* Microgram, *ng* nanogram.

Indoor air quality in photocopier centers shows the presence of high levels of particulate matter in these work places. The levels of other air quality parameters were within permissible limits in all the units.

### Cohort characteristics

Demographic data of the subjects studied are presented in Table 5. No significant differences were observed between photocopier operators and healthy control subjects in age, gender distribution or Body Mass Index (BMI).

**Table 5:** Socio economic status and lifestyle of the study subjects

**Demographics**	**Control**	**Photocopier workers**	**p value**
N	43	81	
Male (%)	30 (70)	64 (79)	0.253*
Age Mean (S.D)	31.7 (7.4)	32.6 (7.1)	0.467**
Monthly income(Rs)			
≤3000	1 (2)	2 (3)	0.047†
3001–10000	27 (63)	61 (75)	
10000–20000	5 (12)	13 (16)	
>20000	10 (23)	5 (6)	
BMI Mean (S.D)	24.2 (4.0)	24.9 (6.2)	0.513**
Current smokers (%)	8 (19)	30 (37)	0.041*
Pack years Mean (S.D)	2.0 (1.8)	2.9 (4.6)	0.510**
Cumulative exposure (1000 s of hours)			
10 – 20		13	
21 – 30		30	
31–40		13	
41 – 70		15	
Alcohol use	11 (26)	30 (37)	0.232

* - Chi square test; ** - Students *t* test; † − Fisher’s exact test.

*BMI* Body mass index; Pack years = (Average number of cigarettes per day/20) × number of years smoked.

Cumulative exposure = No. of working hours/day × No. of working days/week × No. of years of exposure × 50 weeks/year.

### Prevalence of respiratory symptoms and general health problems

**Figure 1:**
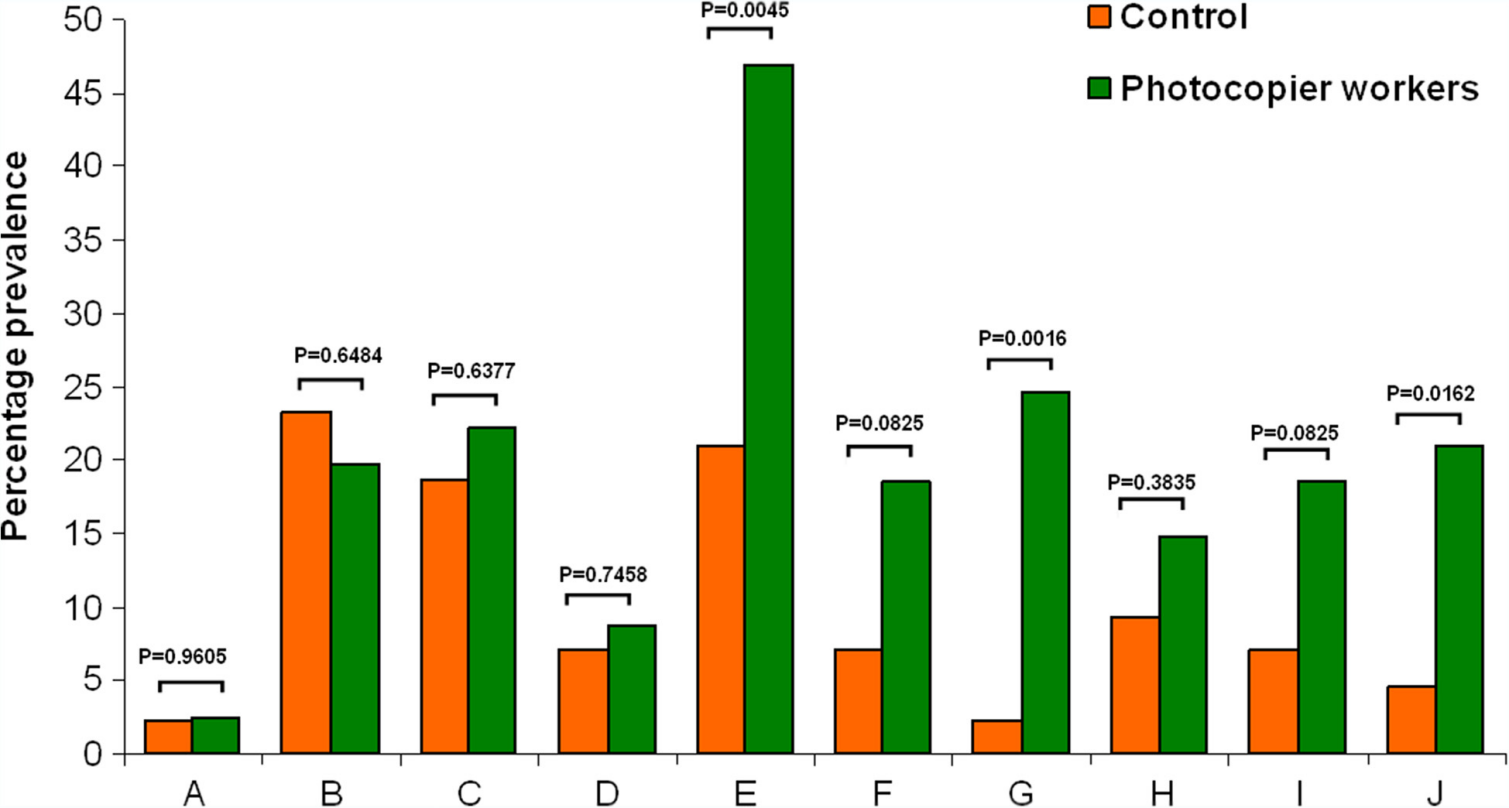
**Symptoms of exposure among the subjects. A** - Skin problems; **B** - Eye problems; **C** - Nose irritation; **D** – Throat pain; **E** – Nasal blockage; **F** – Cough; **G** – Excessive sputum production; **H** – Wheezing; **I** – Allergies; **J** – Breathing troubles.

Prevalence of respiratory symptoms and general health problems among the subjects is presented in Figure 1. Photocopier operators reported significantly higher incidence of nasal blockage (p = 0.003) and breathing troubles (p = 0.03). Excessive sputum production was also reported by a large number of photocopier operators when compared to control subjects (p = 0.004).

### Lung function

Prevalence of respiratory diseases (mild, moderate or severe restriction and/or obstruction) is presented in Table 6. High prevalence of restrictive lung disease (28% among control group and 30% among photocopier operators) was observed. However, no significant difference was observed between the photocopier operators and control subjects in the prevalence of respiratory diseases. A sub group analysis was carried out among non smokers of the study population. No significant difference was observed in the prevalence of lung diseases between non smoking photocopier operators and controls.

**Table 6:** Prevalence of lung diseases

**Groups**	**Control n (%)**	**Photocopier operators n (%)**	**p value**	**Photocopiers n = 46**	**Control n = 29**	**p value**
Restriction	12 (28)	24 (30)	0.841	5 (11)	3 (10)	0.766
Obstruction	4 (9)	8 (10)	0.918	12 (26)	10 (35)	0.305
Restriction & obstruction	2 (5)	4 (5)	0.967	4 (9)	2 (7)	0.780

p values for Chi Square test.

**Figure 2:**
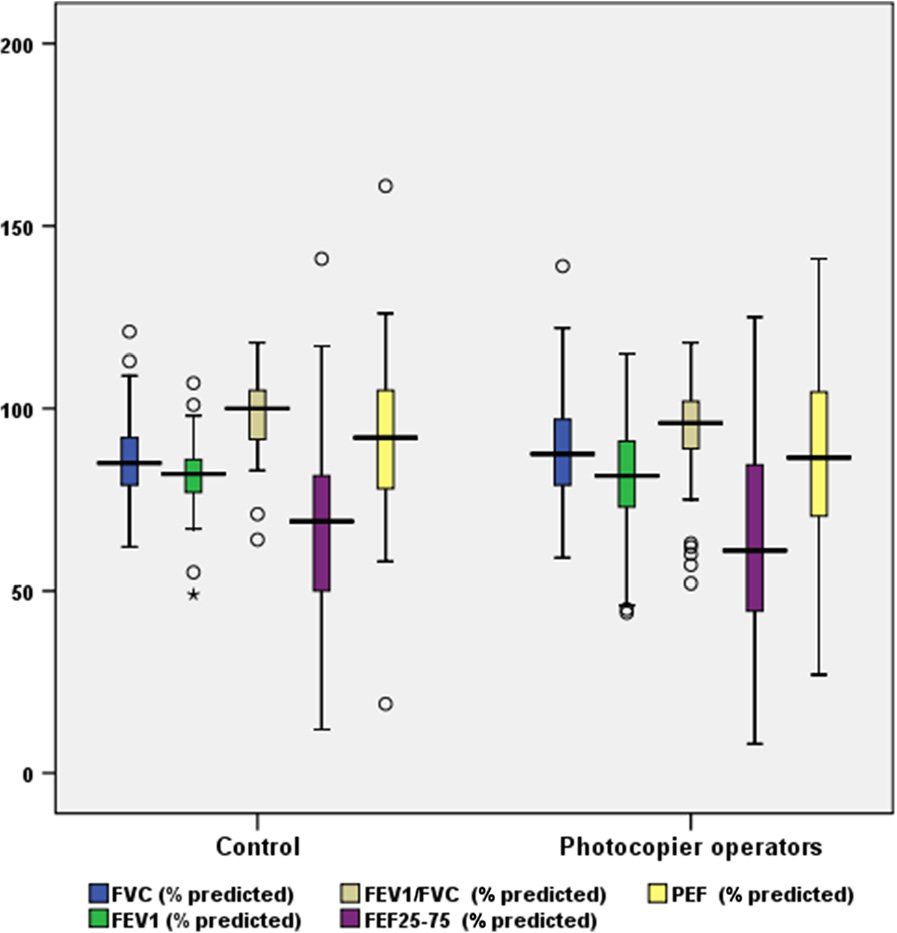
**Lung function parameters of the subjects.** The box plots show the sample median (central vertical line), and the range within which the central 50% of values fall (box length), with the box edges at the first and third quartiles. The whiskers show the range of values, and outsider values are indicated by small “o”s. Far out value is indicated by asterisks.

No significant difference was observed in % predicted values of forced vital capacity (FVC), forced expiratory volume in 1 second (FEV_1_), FEV_1_/FVC, peak expiratory flow (PEF) and maximal mid expiratory volume (FEF_25-75_) between the photocopier operators and control subjects (Figure 2). Sub group analysis among non smokers did not reveal any significant difference between the two groups.

### Confounders and lung function

All the subjects of the study used liquefied petroleum gas as household cooking fuel eliminating the interference of biomass fuel smoke. Cigarette smoking is a confounder in occupational exposure studies. However, lung function among smokers was not significantly different between the photocopier operators and healthy control subjects (data not shown). An interaction variable was created between pack years smoked and years of exposure. This variable did not show correlation with any of the lung function indices.

### Hematological status

Table 7 shows the haematological status of the study subjects. Hematocrit, Mean Corpuscular Volume (MCV) and Red cell Distribution Width (RDW) were significantly higher among photocopier operators when compared to controls. Mixed cells (comprising eosinophils, basophils and monocytes) were significantly lower among photocopier operators than control subjects. No difference was noticed in the levels of circulating total white blood cells, lymphocytes and neutrophils between the two groups of subjects. Hematological status among non smoking photocopier operators and controls was not different from that of photocopier operators and controls. This suggests that smoking does not significantly influence hematological status in this study population.

**Table 7:** Hematological parameters

**Marker**	**Control n = 43**	**Photocopier operators n = 81**	**p value**	**Non smokers**		
				**Control n = 29**	**Photocopier operators n = 46**	**p value**
WBC (10^3^/μL)	7.1 (5.8 – 8.2)	6.6 (5.6 – 7.7)	0.216	7.1 (5.5 – 8.9)	7.0 (5.4 – 8.2)	0.358
RBC (10^6^/μL)	5.1 (4.6 – 5.8)	5.5 (4.9 – 6.0)	0.082	5.0 (4.4 – 5.5)	5.6 (4.7 – 6.1)	0.028
Hemoglobin (g/dL)	15.1 (12.1 – 16.6)	16.0 (13.9 – 17.0)	0.085	13.5 (11.5 – 15.9)	15.5 (13.3 – 16.6)	0.065
Hematocrit (%)	44.1 (37.4 – 48.0)	46.9 (43.8 – 50.2)	0.015	39.5 (35.5 – 45.6)	45.4 (38.4 – 49.0)	0.017
MCV(fL)	84.0 (79.6 – 87.8)	88.4 (83.5 – 91.8)	0.002	83.1 (75.9 – 86.7)	85.3 (80.9 – 90.5)	0.047
MCH(pg)	28.7 (25.6 – 30.5)	29.3 (27.8 – 31.6)	0.064	28.5 (24.2 – 30.4)	28.7 (26.8 – 30.5)	0.485
MCHC (g/dL)	34.0 (32.5 – 35.5)	33.8 (32.0 – 35.0)	0.730	34.0 (31.9 – 35.8)	33.6 (31.3 – 34.9)	0.474
Platelets (10^3^/μL)	255 (221 – 315)	267 (221 – 303)	0.914	281 (226 – 318)	259 (210 – 316)	0.334
Lymphocytes (10^3^/μL)	2.2 (1.9 – 3.0)	2.3 (1.9 – 2.6)	0.713	2.2 (1.8 – 2.9)	2.4 (1.9 – 2.7)	0.830
Neutrophils (10^3^/μL)	3.7 (2.9 – 4.5)	3.7 (3.1 – 4.8)	0.928	3.9 (3.3 – 5.8)	4.0 (3.4 – 5.1)	0.694
Mixed cells (10^3^/μL)	0.8 (0.7 – 1.1)	0.7 (0.5 – 0.9)	0.004	0.8 (0.6 – 1.0)	0.7 (0.5 – 0.9)	0.062
RDW (fL)	42.7 (40.9 – 44.4)	44.5 (42.5 – 47.0)	<0.001	42.4 (40.8 – 43.5)	44.1 (46.5)	0.006
PDW (fL)	12.3 (11.3 – 13.9)	12.1 (11.3 – 13.2)	0.829	12.2 (11.2 – 13.9)	12.4 (11.6 – 13.1)	0.693
MPV (fL)	9.8 (9.4 – 10.7)	9.9 (9.4 – 10.4)	0.920	9.8 (9.4 – 10.5)	10.1 (9.7 – 10.4)	0.271
PLCR (%)	23.9 (20.1 – 29.6)	25.0 (19.8 – 28.5)	0.944	23.3 (20.0 – 28.9)	26.5 (23.0 – 28.1)	0.274

*RBC* Red blood cells, *MCV* Mean corpuscular volume, *MCH* Mean corpuscular hemoglobin, *MCHC* Mean corpuscular hemoglobin concentration, *RDW* Red cell distribution width, *PDW* Platelet distribution width, *MPV* Mean platelet volume, *PLCR* Platelet large cell ratio, *μL* Microlitre, *dL* Deciliter, *fL* (femtolitre), *pg* (picogram), p value for Mann Whitney test.

### Protein levels and oxidative status

Serum protein levels, oxidative status and inflammatory markers are shown in Table 8. No significant difference was found in serum protein levels between the two groups. However, albumin: globulin ratio was found to be significantly altered in the photocopier operators (1.1) when compared to controls (1.5).

**Table 8:** Markers of oxidative-inflammatory status

**Marker**	**Control n = 43**	**Photocopier operators n = 81**	**p value**	**Non smokers**		
				**Control n = 29**	**Photocopier operators n = 46**	**p value**
Total protein (g/L)	68.9 (63.0 – 76.3)	72.4 (66.1 – 75.3)	0.167	68 (60.1 – 73.0)	72 (67.7 – 74.8)	0.048
Albumin (g/L)	39.6 (36.7 – 44.9)	37.1 (33.1 – 40.9)	0.011	38.8 (34.6 – 42.3)	36.5 (32.9 – 41.0)	0.228
Globulin (g/L)	27.1 (20.4 – 35.8)	34.0 (28.6 – 39.4)	0.015	27.9 (20.4 – 35.5)	34.0 (29.0 – 39.3)	0.042
FRAC (mM)	1.5 (0.8 – 1.9)	1.0 (0.8 – 1.4)	0.010	1.6 (0.8 – 2.0)	1.0 (0.7 – 1.4)	0.008
TBARS (μM)	1.4 (1.0 – 1.9)	2.7 (2.1 – 3.5)	<0.001	1.4 (0.8 – 1.7)	2.8 (2.1 – 3.4)	<0.001
8-Isoprostane (pg/mL)	41.7 (23.6 – 52.8)	46.4 (31.7 – 65.4)	0.146	35.4 (20.5 – 51.5)	45.5 (24.6 – 67.5)	0.159
CRP (μg/mL)	1.4 (0.6 – 2.6)	0.9 (0.4 – 1.8)	0.083	1.7 (1.0 – 2.8)	1.0 (0.4 – 2.0)	0.051
ICAM-1 (ng/mL)	113 (97 – 165)	187 (104 – 266)	0.015	113 (99 – 165)	135 (101 – 239)	0.356
LTB_4_ (ng/mL)	13.0 (7.6 – 40.1)	81.9 (22.3 – 159.1)	<0.001	9.2 (6.5 – 15.9)	78 (12 – 169)	<0.001
CC-16 (ng/mL)	7.3 (2.7 – 28.3)	7.8 (3.3 – 33.8)	0.705	7.8 (2.7 – 36.1)	7.1 (2.9 – 32.3)	0.908
ECP (ng/mL)	135 (80 – 207)	213 (118 – 307)	0.009	128 (80 – 207)	215 (147 – 327)	0.015
IL-8 (pg/mL)	17.4 (10.6 – 18.6)	18.1 (17.1 – 20.7)	0.001	16.4 (8.6 – 17.8)	18.2 (16.7- 21.1)	0.002

*FRAC* Ferric Reducing Antioxidant Capacity, *TBARS* Thiobarbituric acid reactive substances, *CRP* C-reactive protein, *ICAM-1* Intercellular Adhesion Molecule 1, Leukotriene B_4_ – Leukotriene B4, *CC-16*, Clara cell protein, *ECP* Eosinophilic Cationic Protein, *IL-8* Interleukin 8, *pg* Picogram, *μg* Microgram, *ng* Nanograms, *mM* Millimoles, *μM* Micromoles, p values for Mann Whitney test.

Serum thiobarbituric acid reactive substances (TBARS) concentration was significantly higher (p < 0.001) among the photocopier operators when compared to the controls. Serum total ferric reducing antioxidant capacity was found to be significantly lower (p = 0.001) among operators in photocopier centers when compared to healthy controls.

Plasma ICAM-1 (p = 0.015), leukotriene B_4_ levels (p < 0.001), ECP (p = 0.009) and IL-8 (p = 0.001) were significantly higher in the photocopier operator group in comparison to the control group. Other inflammatory markers were not significantly different between the two groups.

### Lung function and inflammatory markers

Plasma CC-16 was found to have positive association with % predicted FVC (r = 0.195, p = 0.04). Plasma CRP levels were also found to have a negative correlation with % predicted FVC (r = −0.261, p = 0.005) and % predicted FEV_1_ (r = −0.221, p = 0.019). No significant relationship was found between lung function and other inflammatory biomarkers.

### Smoking status and inflammatory biomarkers

Smoking status was found to have a positive correlation with plasma levels of CC-16 (r = 0.167, p = 0.033). Interaction between smoking and cumulative hours of exposure was studied on the inflammatory markers. This interaction was not found to alter the effects of photocopier exposure on the different markers assessed. Among non smokers, levels of inflammatory markers were similar to that observed in the combined group suggesting no appreciable influence of smoking.

## Discussion

Ambient air quality in photocopier centers has not been previously reported in India. Levels of PM_2.5_ and PM_10_ were about 2–3 fold higher than the permissible limits during working hours. Even background PM_2.5_ and PM_10_ levels were higher than the permissible levels. Similar results have been reported by many investigators around the world over the last decade [39-47]. Emissions of nanoparticles from photocopiers have also been recently reported [48]. Long term exposure to particulate matter has been associated with cardiovascular disease by the mechanisms of systemic inflammation, direct and indirect coagulation activation and direct translocation into systemic circulation. Respiratory diseases are also exacerbated by exposure to PM by creating oxidative stress and inflammation [49-51].

The levels of other ambient air quality parameters such as carbon monoxide, nitrogen dioxide, ozone, sulphur dioxide, lead, arsenic, nickel and ammonia. Benzene and benzo(a)pyrene were also within permissible levels. The levels of benzene, benzo(a)pyrene were also within limits in spite of the presence of many VOC sources in these photocopy centers. Previous studies [52-54], also found that benzene, toluene, ethylbenzene, xylenes (BTEXS) and styrene were below the occupational exposure threshold guidelines in photocopier centers. All the photocopier centers included were open air buildings where one wall was replaced by retractable shutters, open for ventilation throughout the business hours. In spite of the improved air circulation in the photocopier centers, the very high levels of particulate matter in these centers are an area of concern.

Photocopier operators have a high prevalence of respiratory problems such as nasal blockage, breathing troubles and excessive sputum production when compared to control subjects. This might be due to the high particulate matter exposure. Particulate matter might contribute to increased respiratory symptoms and chronic nasal and paranasal sinus problems [55,56].

This is the first study on lung function in photocopier operators. The photocopier operators assessed in the present study worked with imported second hand copiers, which have been, since, banned in India.

The high prevalence of restrictive lung diseases among the both the groups of subjects could not be explained in the context of the present study. No data is available on the prevalence of lung diseases in this geographical area/ethnic group to compare the data. A larger population study might throw light on the causes for this high prevalence.

Lung function parameters did not show any significant differences between the photocopier operators and healthy control groups. Studies on pulmonary function among operators in toner manufacturing units in Japan also reported similar results [2,22,23].

In the present study, the percentage of smokers was significantly higher among photocopier operators group when compared to controls. However, smoking status did not significantly influence lung function and inflammation of the photocopier operators and control subjects. This might be due to the very few pack years smoked between the cohorts.

Hematocrit is the volume of red blood cells expressed as a percentage of a given volume of whole blood. Higher levels of hematocrit, were associated with an increased risk of developing heart failure in a long-term follow-up study [57]. The high levels of hematocrit among photocopier operators also signify increased cardiovascular risk which is rational considering the high particulate matter exposure.

Red blood cell distribution width (RDW) is a numerical measure of the size variability of circulating erythrocytes and is routinely reported as a component of complete blood count in the differential diagnosis of anemia. RDW has been recently reported to be a strong and independent predictor of adverse outcomes in the general population [58,59]. High RDW might also reflect a general inflammatory state causing ineffective erythropoiesis in addition to vascular disease progression [60]. High RDW among the photocopier operators may be due to chronic photocopier exposure and reflect their inflammatory state.

Mixed cells comprising of eosinophils, basophils and monocytes were significantly lower among the photocopier operators. All these cells are involved in the inflammatory response to tissue injury. The significant decrease in mixed cell population might be due to the migration of these cells to the airways, the site of injury caused by chronic exposure to pollutants. A decrease in circulating monocytes and increase in migration of monocytes to inflammatory sites upon exposure to ambient air pollution have been observed by earlier studies [61].

Albumin levels were significantly reduced in photocopier operators suggesting inflammation. When albumin concentration is reduced, other risk factors associated with both inflammation and cardiovascular risk is increased [62]. An increase in serum globulin levels may be reflective of a mild systemic immune response [63]. In the present study, significantly decreased albumin and increased globulin among photocopier operators might be caused by inflammation and systemic immune response.

Oxidative stress followed by inflammation is thought to be central in the mechanisms of action for the health effects of PM [64]. Serum TBARS was significantly increased in the photocopier operators when compared to the unexposed controls. This is in tune with the high serum TBARS among photocopier operators already reported [13]. However, levels of 8-Isoprostane did not show significant differences between the two groups. TBARS may be a more sensitive biomarker than 8-isoprostane and may be a useful tool for investigating air pollution-related oxidative stress as discussed by [65].

In harmony with the above results, total serum ferric reducing antioxidant capacity was significantly reduced among the photocopier operators when compared to the healthy control group suggesting increased oxidative stress. The results of this study show that exposure to emissions from photocopiers on a regular basis causes oxidative stress analogous to the effects of several air pollutants, diesel exhaust and cigarette smoke [66-68].

The lack of significant increase in CRP levels among photocopier workers when compared to control is mystifying, given the significant rise in other inflammatory markers. The insignificant increase in CRP levels among photocopier workers may be in view of the fact that particulate matter is the primary pollutants emitted by photocopiers. No consistent rise has been observed in CRP levels after exposure to particulate matter [69].

CRP levels have been strongly and independently associated with lung function by quite a few studies over the years [70-72]. In concurrence, in the present study, C Reactive Protein was found to be the best indicator of pulmonary function among the inflammatory markers assayed in the present study. Significant negative correlations with percent predicted FVC and FEV_1_ were observed. The lack of elevation in CRP was in accordance with the lack of lung function impairment.

Intercellular adhesion molecule (ICAM)-1 is a cell adhesion molecule expressed by several cells including leukocytes and endothelial cells. Soluble ICAM-1 (sICAM-1) is a form of ICAM-1 found in plasma. sICAM-1 levels are elevated in cardiovascular diseases, autoimmune disorders, cancer and acute lung injury [73,74]. Concentration of sICAM-1 is an indicator of coronary heart disease [75]. The significant increase in plasma ICAM-1 of photocopier operators may be related to systemic inflammation caused by exposure to emissions from photocopiers on a routine basis.

Significant rise in leukotriene B_4_ levels of the photocopier exposed subjects in the present study is suggestive of inflammation. Further, LTB_4_ has also been associated with the progression of atherosclerosis. The main effect of the pro-inflammatory eicosanoid leukotriene B_4_ is related to the inflammatory response and it facilitates capillary extravasation and a maintained immune activation within the atherosclerotic lesions [76,77].

Clara cells can modulate immune responses by secretion of both pro- and anti-inflammatory factors. CC-16 is a natural immune-regulator protecting the respiratory tract from unwanted inflammatory reactions [78]. CC-16 levels in serum increase when lung epithelium permeability is adversely affected by air pollutants or other lung toxicants. On the contrary, reduced levels of CC-16 in lung lavage fluid occur in several lung disorders, probably due to a decrease in the production of CC-16 as a consequence of a depletion of Clara cells [79]. In the present study, neither significant increase nor significant decrease was observed in the plasma levels of CC-16 of photocopier operators indicating insignificant effects of the PM on the lung epithelium which is surprising. However, changes in CC-16 and CRP levels were not observed after in traffic exposure to PM_2.5_ and PM_10_[80].

ECP is the best known of the eosinophil proteins, assessed and used extensively. It is transported and stored in the mature eosinophil granules at a high rate. The majority of ECP is released after the cell has left the circulation. Several types of inflammatory stimulations have been shown to cause eosinophil degranulation [81]. In the present study, elevation of plasma ICAM-1 and LTB_4_ observed in the photocopier exposed subjects, might have contributed to the elevated levels of plasma ECP in the subjects. This provides further evidence of inflammation due to exposure to emissions from photocopiers.

IL-8, a proinflammatory cytokine, was also significantly higher among photocopier operators [82,83]. Increased serum levels of IL-8 are correlated with an increased risk of cardiovascular disease or acute cardiovascular events [84]. Thus, in the present study, high plasma IL-8 levels signify inflammation among photocopier operators.

Oxidative stress and inflammation are strongly connected and either one can lead to the other. It is likely that the pro-oxidant potential of ambient particles determine their ability to activate pathways that lead to pro-oxidant and pro-inflammatory effects in the vasculature and promotion of atherosclerosis [85]. Chronic exposure to emissions from photocopiers may lead to similar consequences, due to the chronic particulate matter exposure in these units.

## Conclusions

Long term exposure to emissions from photocopiers was not associated with decreased lung function. However, photocopier exposure was associated with high oxidative stress and inflammation, leading to higher risk of atherosclerosis and cardiovascular diseases.

## Abbreviations

8 IP: 8-Isoprostane; CC-16: Clara cell protein; CRP: C reactive protein; ECP: Eosinophilic cationic protein; ELISA: Enzyme linked immunosorbent assay; FRAC: Ferric reducing antioxidant capacity; FVC: Forced vital capacity; FEV1: Forced expiratory volume in 1 second; ICAM-1: Inter cellular adhesion molecule 1; IL-8: Interleukin 8; LTB4: Leukotriene B_4_; TBARS: Thio barbituric acid reactive substances.

## Competing interests

The authors have not over the past three years had any financial relations with organizations that might have an interest in the submitted work. The authors hereby declare no relationships or activities that could appear to have influenced the submitted work.

## Authors’ contributions

NE conceptualized and designed the experiments, recruited majority of the subjects, carried out pulmonary function tests, conducted majority of experiments, analyzed the data, performed statistical analysis and took the lead on writing the manuscript. VK collected samples, carried out experiments, helped in interpretation of results and revising the manuscript. BV contributed to manuscript preparation. All authors read and approved the final manuscript. JGP contributed to the design of the study, supervised the study and helped with revising the manuscript.
